# Facile construction of the aerogel/geopolymer composite with ultra-low thermal conductivity and high mechanical performance

**DOI:** 10.1039/c7ra12041a

**Published:** 2018-01-09

**Authors:** Yajun Huang, Lunlun Gong, Yuelei Pan, Congcong Li, Ting Zhou, Xudong Cheng

**Affiliations:** State Key Laboratory of Fire Science, University of Science and Technology of China Hefei Anhui 230027 PR China chengxd@ustc.edu.cn

## Abstract

In this work, we have successfully prepared a lightweight, highly hydrophobic and superb thermal insulating aerogel/geopolymer composite by a sol–gel immersion method. After silica aerogel was impregnated, the composite exhibited nano-porous structures. Moreover, scanning electron microscopy observations revealed that the aerogel particles were tightly anchored on the geopolymer surface. With several excellent properties (bulk density: 306.5 g cm^−3^, thermal conductivity: 0.0480 W m^−1^ K^−1^ and maximum compressive strength: 0.79 MPa) the as-prepared composite shows great potential to be applied in the thermal insulation field.

## Abstract 2

In this work, we have successfully prepared a lightweight, highly hydrophobic and superb thermal insulating aerogel/geopolymer composite by a sol–gel immersion method.

## Graphical abstract

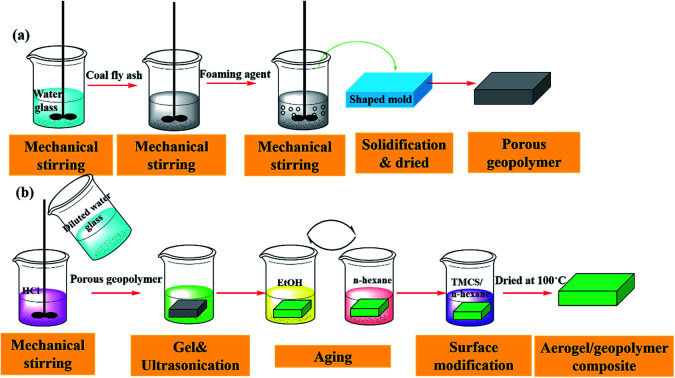

## 1. Introduction

With the development of industrialization, a large number of solid wastes (fly ash, slag, *etc.*) have been produced, which are harmful to the environment and human health. Thus, how to dispose of them becomes a significant subject. The name ‘‘geopolymer’’ was originally proposed by Davidovits^1^ to describe the inorganic aluminosilicate polymers produced by synthesizing natural materials such as metakaolin or industrial solid wastes. An efficient treatment for these solid wastes is the usage as the raw materials of porous geopolymer, which stimulates wide research interest.^2–7^ This method can not only reduce the energy consumption, but also reuse the waste. T. W. Cheng *et al.* have prepared the fire-resistant geopolymer produced by granulated blast furnace slag. They found the geopolymerisation behavior, physical, mechanical properties and fire resistance characteristics were strongly dependent upon the chemical composition in the reaction system.^8^ Wang *et al.* have synthesized the metakaolinite-based geopolymer and investigated its mechanical property. It is found that the mechanical properties of the geopolymers were greatly dependent on the concentration of NaOH solution and the flexural strength, compressive strength, and apparent density of the geopolymer increased along with the increase of NaOH concentration within 4–12 mol L^−1^.^9^ P. Chindaprasirt *et al.* have studied the workability and strength of coarse high calcium fly ash geopolymer. The results revealed that the workable flow of geopolymer mortar was in the range of 110–135%, which was dependent on the mass ratio of sodium silicate and NaOH and the concentration of NaOH solution.^10^ Furthermore, geopolymer also showed potentials in thermal insulating materials, considering its low thermal conductivity.^11–14^ However, the thermal conductivity of this materials failed to meet the high requirements in some occasions. Therefore, the further decrease of its thermal conductivity has been a hot topic and relevant research work should be done.

Silica aerogel, derived from the sol–gel method, has a unique nanometer network. This feature leads to outstanding properties, such as a high specific surface area (500–1200 m^2^ g^−1^), high porosity (80–99.8%), a low density (0.003–0.5 g cm^−3^) and a high thermal insulation value (0.005–0.015 W m^−1^ K^−1^).^15–17^ Whereas, the aerogel's poor mechanical property has greatly limited its application. To improve the mechanical property, aerogel composites were developed by researchers.^18–23^ Gao *et al.* have prepared the lightweight concrete by incorporating hydrophobic aerogel granules into a concrete matrix. The composite showed a thermal conductivity of 0.26 W m^−1^ K^−1^ with an aerogel content of 60 vol%.^24^ Serina Ng *et al.* have used partial replacement of ordinary Portland cement with calcined clay as binder in the aerogel incorporated mortars, decreasing the thermal conductivities and maintaining the mechanical strength.^25^ Hong *et al.* have fabricated the silica aerogel/new porous zirconia ceramic composite materials. The coefficient of thermal conductivity decreased from 0.085 to 0.041 W m^−1^ K^−1^ and the compressive strength increased from 9.20 to 15.4 MPa.^26^ Yang *et al.* have prepared a novel silica aerogel/porous silicon nitride composites by the freeze casting and sol–gel impregnation method. The thermal conductivity decreased from 0.084 to 0.043 W m^−1^ K^−1^ and the compressive strength increased from 4.22 MPa to 7.08 MPa.^27^ These composites all shows improved thermal insulating and mechanical properties in comparison to pure silica aerogel. Therefore, it is believed that the combination of silica aerogel and geopolymer can also endow the prepared composite with favorable mechanical and thermal insulating properties, simultaneously. However, there is little research on aerogel/geopolymer composite. A patent presented by Kim *et al.*^28^ prepared geopolymer–aerogel composite with the thermal conductivity of 0.2 W m^−1^ K^−1^ through a physical method. Nevertheless, the thermal conductivity is relatively high for the requirement of thermal insulation.

In this paper, the solid waste of coal fly ash was used as the raw material for geopolymer. Then, silica aerogel was impregnated in this prepared geopolymer, resulting in the formation of the aerogel/geopolymer composite. The obtained composite showed ultra-low thermal conductivity and high mechanical performance. In detail, the thermal conductivity of composite decreased from 0.0670 to 0.0480 W m^−1^ K^−1^ and the maximum compressive strength increased from 0.45 to 0.79 MPa, when the diluted ratio of water glass is fixed at 3 : 1 in the process of preparing aerogel. The density, thermal insulating and mechanical properties of aerogel/geopolymer samples at different ratio of deionization water (DI) to water glass were also discussed. Owing to its improved mechanical property, the thermal insulating aerogel/geopolymer composite showed potentials in practical applications. In general, the preparation of aerogel/geopolymer composite may provide an efficient strategy for turning waste into wealth.

## 2. Experimental methods

### 2.1 Raw materials

Coal fly ash (100 meshes) was obtained from Hefei Zhong Cheng Thermal Power Co. Ltd (China). The chemical compositions of the fly ash were determined by X-ray fluorescence spectroscopy (XRF-1800; SHIMADU, Tokyo, Japan) and are presented in Table 1.

**Table 1:** Elemental analysis of the fly ash using the XRF technique

Samples	Al_2_O_3_	SiO_2_	Fe_2_O_3_	CaO	K_2_O	TiO_2_	MgO	Na_2_O	SO_3_	Others	Ignition loss
Fly ash	25.35	51.78	4.60	10.04	1.06	1.36	0.36	0.31	4.79	0.35	—

Protein foaming agent was provided by Jinan Hong Tu Chemical Industry Co. Ltd (China). Water glass (wt: 34%, Na_2_O : SiO_2_ = 1 : 3.33) was purchased from the Qingdao Dongyue Sodium Silicate Co., Ltd (China). Other agents including hydrochloric acid (HCl), ethanol (EtOH), *n*-hexane and trimethylchlorosilane (TMCS) were all purchased from Sinopharm Chemical Reagent Co., Ltd, (China).

### 2.2 Preparation process

#### Preparation of porous geopolymer

**Fig. 1:**
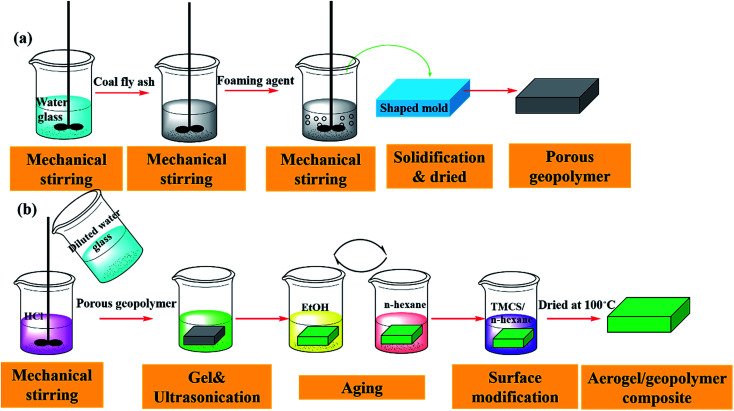
Preparation process of aerogel/geopolymer composite.

As is showed in Fig. 1(a), porous geopolymer was prepared in a rubber mould by adding fly ash (sift through 100 meshes), water glass, water, high efficiency foaming agent of proteins category. The mass ratio of fly ash, water glass, water and foaming agent was 8 : 7 : 2 : 5. Firstly, fly ash and water glass were mixed with a certain amount of water, which were added slowly to obtain a uniform coating mixture. At the same time, the mixture was stirred at 450–500 rpm for 2–3 min. After that, the foaming agent was added into the slurry gradually. The mixture was stirred continuously with speed increasing to 600 rpm slowly and the mixture was stirred for 5–6 min at the speed of 600 rpm. Secondly, the well-mixed slurry was poured into a stainless rubber shaped mold (60 mm × 60 mm × 20 mm). Then, the wet sample was placed at room temperature for 24 h for the natural evaporation of surface water, resulting in its solidification. The samples were moved to humidity chamber for curing. Lastly, the sample was placed at room temperature for 24 h and moved into a drying container.

Preparation of aerogel/geopolymer composite: Fig. 1(b) presents the preparation process of composite. Silica alcogel was prepared through a two-step, acid–base catalyzed sol–gel process. Firstly, the premixed solution, water glass was diluted with different volume of deionized water and hydrolysis was conducted by adding 0.1 M HCl. The sol was stirred for 1 min in room temperature (25 °C). The diluted water glass, serving as base catalyst, was added to the solution to adjust pH to ∼5.0. Then, the porous geopolymer was impregnated with the silica sol under ultrasonic vibration. Wet gel in the porous geopolymer was formed by the condensation process within 10 min at the room temperature (25 °C). The cured silica aquagel/porous geopolymer was aged in EtOH at 45 °C for 6 h. Water in the hydrogel pores can be exchanged by EtOH and EtOH was further replaced by *n*-hexane. The above processes were repeated several times. Surface modification of the gel in the composite was carried out by adding TMCS/*n*-hexane solution with the molar ratio of 1 : 2. The reaction occurred under 45 °C for 6 h. The modification process is also repeated several times. At last, the composite was dried at 100 °C for 5 h.

### 2.3. Characterization

The mass (*m*) and volume (*V*) of the sample were measured and the bulk density was calculated by this two parameters. The specific surface area, pore volume and pore size distribution (PSD) of the composite was determined by Brunauer–Emmett–Teller (BET) analysis, measured at 77 K with a TristarII3020M analyzer. The microstructure and micromorphology of the composite were observed by a field emission scanning electron microscope (SEM, SIRION200, FEI, USA). The thermal stability was tested by the thermal gravimetric and differential thermal gravity (TG-DTG) with the instrument SDT Q600 (TA Instruments, USA). Fourier transform infrared spectroscopy (FTIR, Nicolet 8700, Thermo Fisher Scientific, USA) was employed to investigate the chemical bonding state of the samples. Thermal conductivity (*λ*) is regarded as the important parameters in the field of thermal insulation. Thermal conductivity of the samples were measured on the principle of hot-wire method.^29^*λ* was measured by the thermal conductivity instrument (TC 3000E, Xia xi technology, China). The contact angle (*θ*) was measured with the contact angle instrument (SL200K, USA). Electronic dynamic and static fatigue testing machine (E3000K8953, Instron) was used for compressive strength test. The tested samples have a dimension of 40 mm × 40 mm × 20 mm (length × width × thickness) and the loading rate was set as 1 mm min^−1^ until material failure.

## 3. Results and discussion

**Fig. 2:**
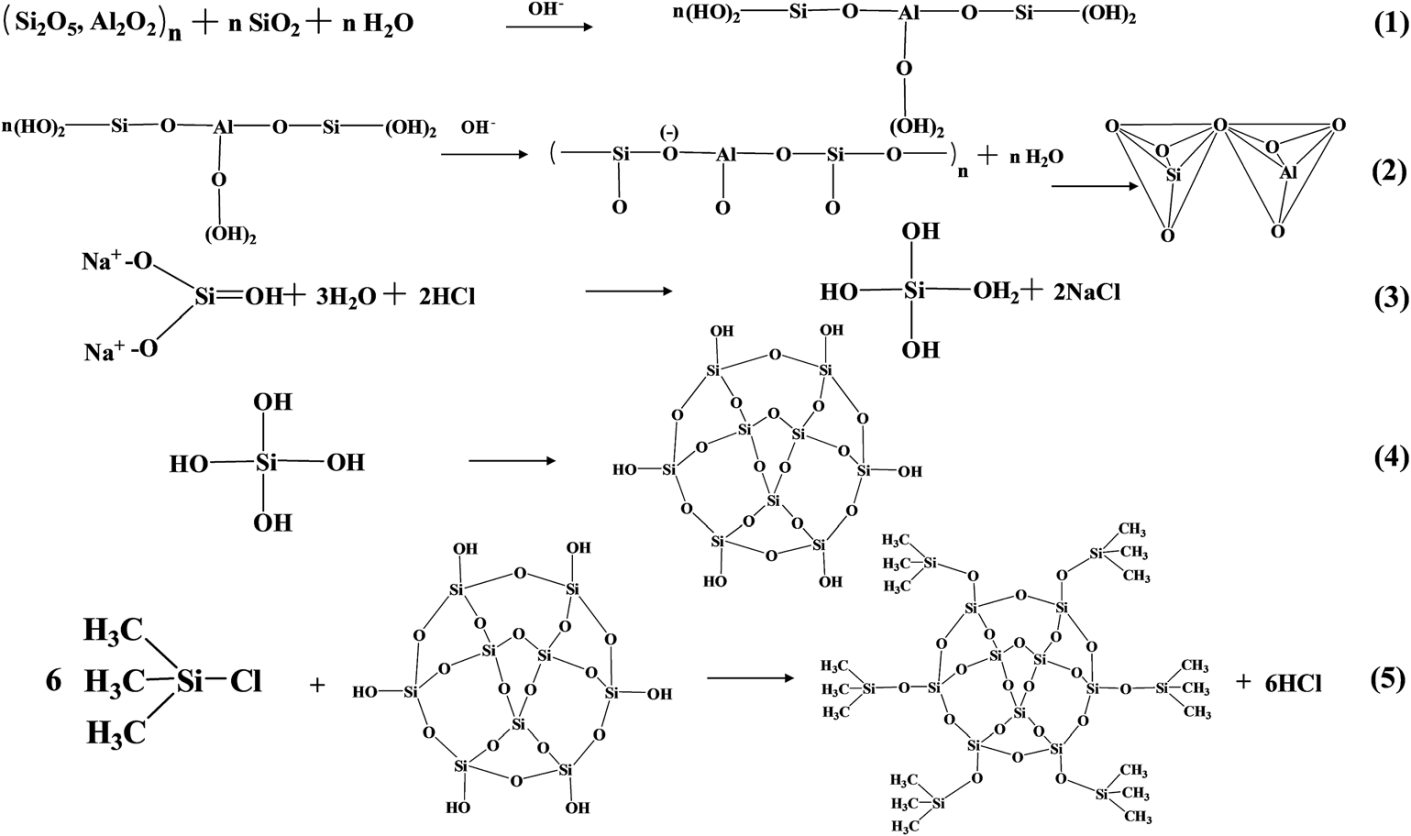
Schematic representation of aerogel/geopolymer formation.

Fig. 2 presents the chemical process of aerogel/geopolymer formation. For preparing the porous geopolymer, the bonds of amorphous Si–O and Al–O on the surface of fly ash were broken firstly in alkaline environment and forming the oligomers (eqn (1)). Then these oligomers become ionic clusters through the process of polycondensation and the network structures is formed (eqn (2)). Silica hydrogels in the porous geopolymer were prepared by the method of sol–gel, at the same time, diluted water glass was utilized as precursor as well as base catalyst. Hydrolysis occurred under acid condition (eqn (3)). Condensation happened in base environment (eqn (4)).

As we know, the liquid within the pores of hydrogel vaporized when ambient pressure drying happened. The capillary pressure (*C*_P_) occurs during evaporation and forced on the pores. And the pores will collapse because of *C*_P_ difference. The solvent exchange and surface modification is necessary for reducing the *C*_P_.^30^ Ethanol and *n*-hexane were utilized as the exchange solvents. During the period of surface modification, ≡Si–OH groups on the surface of the gel skeletons were substituted by –Si–(CH_3_)_3_ groups originated from TMCS (eqn (5)). There is no chemical reaction between silica aerogels and porous geopolymer. Finally, the silica aerogel were introduced into porous geopolymer successfully after drying.

**Fig. 3:**
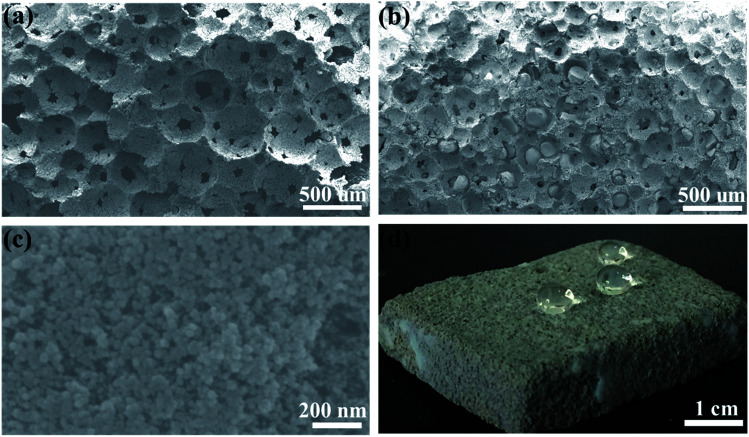
SEM images of: (a) the porous geopolymer, (b) aerogel/geopolymer composite and (c) the magnification of the impregnated aerogel. (d) Digital photo of aerogel/geopolymer composite during the hydrophobicity test.

Morphology of geopolymer and the aerogel/geopolymer were investigated by SEM test and their corresponding images are displayed in Fig. 3. The 3-D interconnected porous structure of geopolymer is clearly shown in Fig. 3(a) and lots of spherical pores with no preferred orientation are well distributed on the surface of geopolymer. When the silica aerogel is added, most of the pores in geopolymer are occupied. Then, decrease in the pore size is observed in Fig. 3(b) and the protrusions with smooth surface is attributed to the impregnated silica aerogel compared to Fig. 3(a), suggesting the successful introduction of silica aerogel. The magnification view of silica aerogel in the pores of geopolymer is exhibited in Fig. 3(c). It is obvious that the silica aerogel is composed of nanoparticles and nanopores. Fig. 3(d) is the digital photo of aerogel/geopolymer composite during the hydrophobicity test. After the dripping of water, the spherical droplets are observed on its surface. This suggests the excellent hydrophobicity of the aerogel/geopolymer composite.

**Fig. 4:**
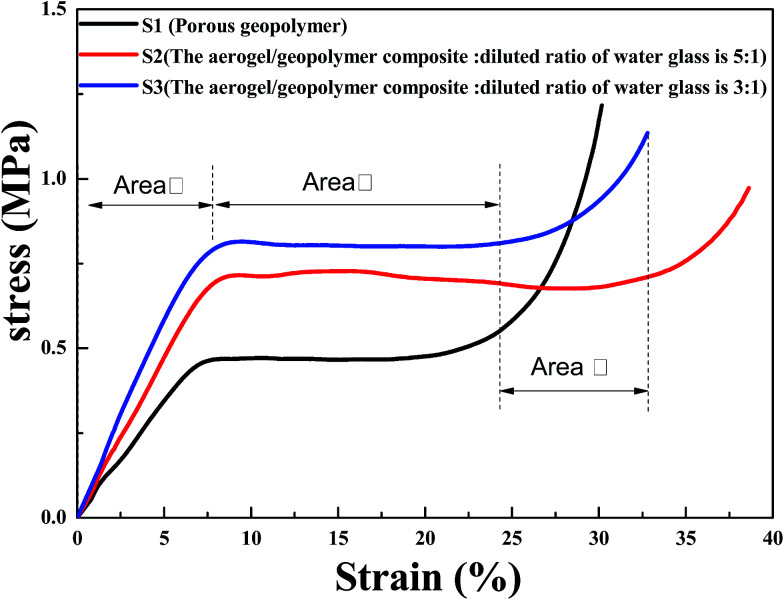
The compressive stress–strain curve of the samples: (S1) porous geopolymer; (S2) the aerogel/geopolymer composite (diluted ratio of water glass is 5 : 1); (S3) the aerogel/geopolymer composite (diluted ratio of water glass is 3 : 1).

Fig. 4 is the compressive stress–strain curves of porous geopolymer, aerogel/geopolymer composite (diluted ratio of water glass: 5 : 1 and 3 : 1). It was observed that all of the stress–strain curves could be divided into three different stages according to slope of the curve. For the S3 curve, aerogel/geopolymer composite contacts with sensors firstly at low strain and the stress increases with the increment of strain (Area I). Then the composite is crushed and the stress is almost unchanged with the increase of strain because of its high porosity (Area II). Lastly, the material is compacted and the stress increases rapidly as the strain increases (Area III). The maximum compressive stress is the point where the material stress stays unchanged as the strain increases. It was found that the mechanical properties of porous geopolymer are improved after impregnating with silica aerogel. The high porosity may lead to an exponential decrease in strength compared to the total dense geopolymer. The structure strength of aerogel/geopolymer composite was enhanced due to the aerogel's occupying in pores, which can bear greater loads. As a result, the improved mechanical property of the composite was achieved. The curves of S2 and S3 show the least and largest of maximum compressive strength of the composites respectively, which is attributed to different diluted water glass when preparing the silica aerogel. Compared with that of porous geopolymer, their compressive strength increase by 57.8 and 76.2%, respectively. With the variation in diluted ratio of water glass, the maximum compressive strength of the composite increases from 0.71 to 0.79 MPa.

**Fig. 5:**
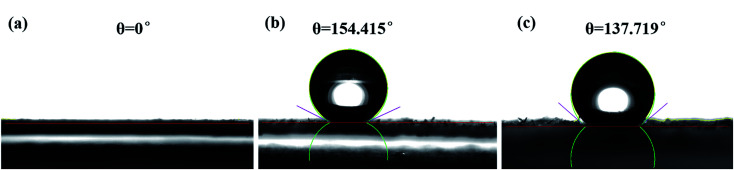
Contact angle of (a) geopolymer; (b) silica aerogel; (c) the aerogel/geopolymer composite.

The silica aerogel in the composite become hydrophobic after surface modification. For the ≡Si–OH groups was substituted by methyl groups from TMCS. The effect of surface modification was studied by the water contact angle. The contact angle (*θ*) was measured with the contact angle instrument and the related results are shown in Fig. 5. The original sample of geopolymer shows a contact angle of 0°, suggesting its hydrophilcity. After the impregnation of silica aerogel, the contact angle of the composite reaches to 137.719°, indicating its hydrophobicity. This obvious difference is mainly due to the introduction of the hydrophobic silica aerogel.

**Fig. 6:**
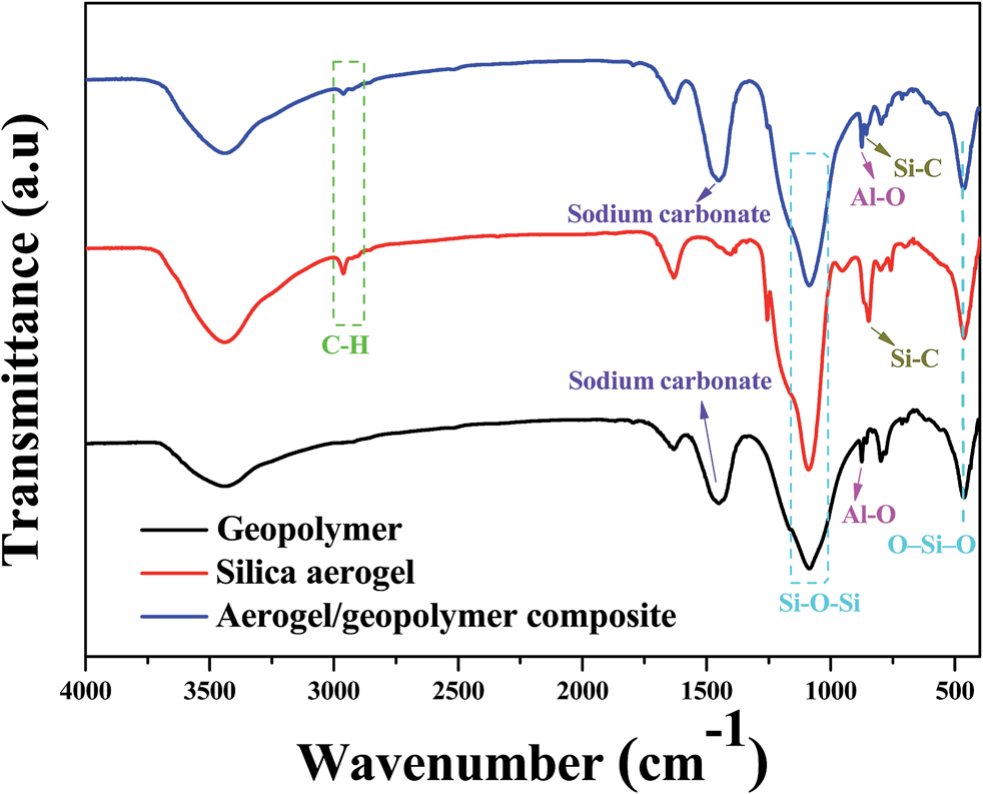
FTIR spectra of geopolymer, silica aerogel and the aerogel/geopolymer composite.

FTIR spectra of geopolymer, silica aerogel and their composite are exhibited in Fig. 6. In the spectrum of silica aerogel, the peak at 2962 cm^−1^ and 850 cm^−1^ can be attributed to –CH_3_ groups and the Si–C bonds, respectively.^31^ The appearance of this two peaks is also observed in the result of the composite, which are indicative of the hydrophobicity of samples. In the spectrum of geopolymer in Fig. 6, the band at 870 cm^−1^ can be assigned to Al–O symmetric stretching in tetrahedral, which is also observed for the composite.^32^ Besides, the band at about 1460 cm^−1^ is related to the presence of sodium carbonate *via* the reaction of alkali metal hydroxide with atmospheric CO_2_.^33^ These two peaks are clearly presented in the spectrum of the composite, which suggests the existence of geopolymer. In addition, the peaks at 1085 and 460 cm^−1^ correspond to Si–O–Si stretching vibration and O–Si–O bending mode, respectively.

**Fig. 7:**
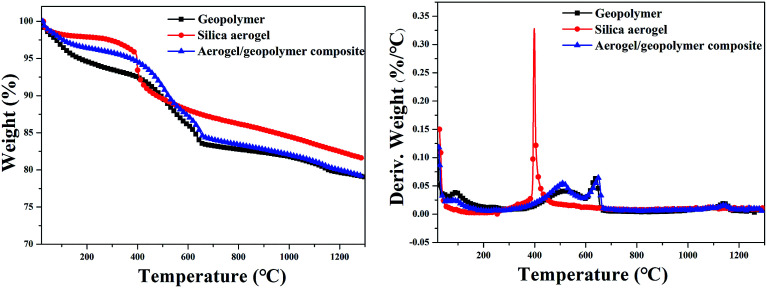
TG/DTG curves in air atmosphere for geopolymer, silica aerogel and aerogel/geopolymer composite.

Thermal decomposition process of geopolymer, silica aerogel and their composite under air were investigated by TG tests and the corresponding results are given in Fig. 7. From the TG curves, the weight loss between 40–200 °C may be assigned to the removal of water. These three samples exhibit high solid residue at 1300 °C and the composite shows similar process with the pristine geopolymer. There is an obvious difference between this two processes. At the early stage, the composite shows higher residue than the pristine geopolymer, which may be attributed to the presence of silica aerogel. A obvious peak at a temperature of ∼415 °C is observed in the DTG curves of silica aerogel, which is mainly caused by the oxidation of methyl groups.^31^ However, this characteristic peak of silica aerogel is not found in the DTG curves of the composite, which may be due to the small quality of aerogels compared to geopolymer.

**Fig. 8:**
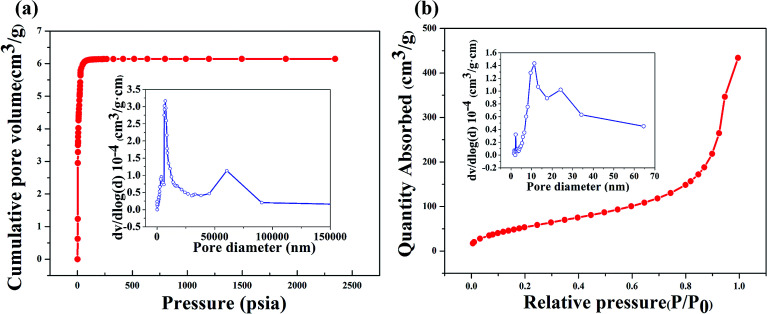
Adsorption isotherms and pore size distribution of: (a) porous geopolymer by mercury porosimetry; (b) aerogel/geopolymer composites by nitrogen adsorption instrument.

Fig. 8(a) shows the N_2_ adsorption isotherms and pore size distributions (PSD) of the porous geopolymer, which exhibits a multimodal pore size distribution with peaks at about 7.5 and 60 μm. As is seen in Fig. 8(b), the PSD of the composite reveals three peaks at 2, 11 and 24 nm. The mean pore size of the porous geopolymer and the composite are 15.80 μm and 18.20 nm, respectively. It is obvious that the size of aperture decreases greatly after the impregnation of silica aerogel. The BET surface areas of the porous geopolymer and the silica aerogel/geopolymer composite are calculated as 7.81 and 212.62 m^2^ g^−1^, respectively.

**Fig. 9:**
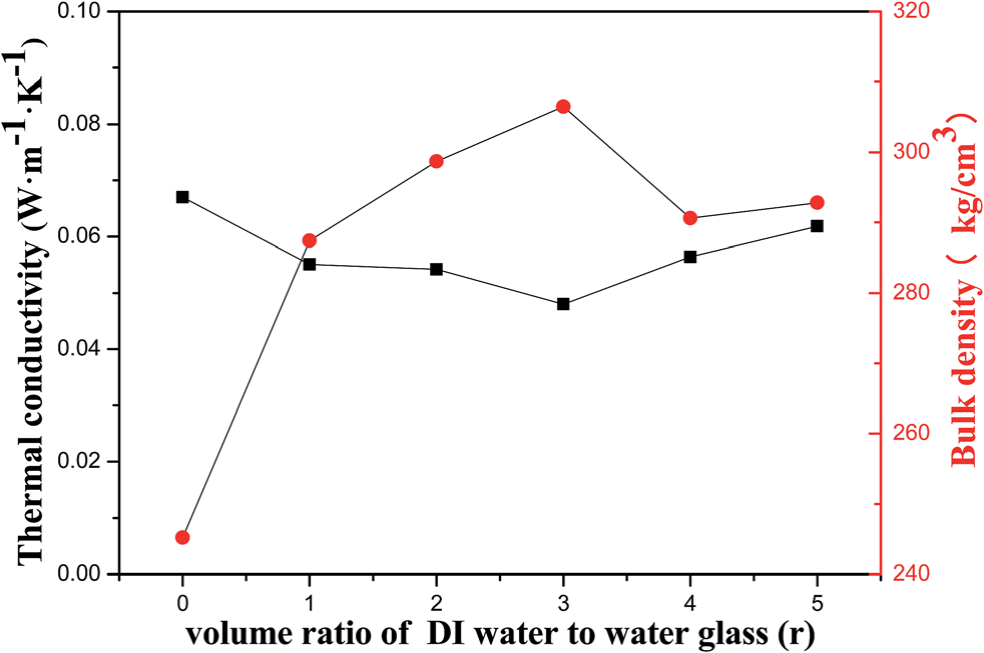
Effect of diluted ratio of water glass on thermal conductivity and bulk density.

The contact angel, thermal conductivity, and bulk density of porous geopolymer and the composites prepared with different diluted ratio of water glass are summarized in Table 2. The sample of porous geopolymer exhibits a thermal conductivity and a bulk density of 0.067 W m^−1^ K^−1^ and 245.2 g cm^−3^, respectively, and the composite with water glass diluted ration of 3 : 1 shows a ultra-low thermal conductivity of 0.048 W m^−1^ K^−1^. Approximately 28% decrease in thermal conductivity is obtained. This can be assigned to the incorporation of silica aerogel, whose thermal conductivity ranges 0.01–0.02 W m^−1^ K^−1^. The mean free path of aerogel (∼50 nm) is smaller than that of air (∼69 nm). Then, air molecules can hardly pass through the aerogel. In the composite, air in the pore regions of porous geopolymer can be replaced by the silica aerogel, resulting in a decrease in thermal conductivity and making the material a better thermal insulator than air.^34^Fig. 9 presents the thermal conductivity and bulk density of the samples with different diluted ratio of water glass. The diluted ratio of water glass is denoted as *r*. It is observed that the thermal conductivity of the composites is obviously smaller and the bulk density is higher than these of the original materials. The declining degree of thermal conductivity of composites mainly depends on two aspects: aerogel content in porous geopolymer, and thermal conductivity of pure aerogel. It can be seen that the composites with highest bulk density can be obtained when the *r* = 3, which means the maximal content of aerogel in porous geopolymer among the samples. At this point, the aerogel content is the dominant factor that contribute to the composites with lowest thermal conductivity. It is also observed that the difference in bulk density is minor when the *r* changes from 4 to 5, which indicates that the difference of aerogel content in this two composites is close. And it is the reason that the thermal conductivity of aerogel lead to the difference in thermal conductivity. The volume would shrinkage and the pore diameter of aerogel would diminish due to the pore collapse with weak cross-linking when there are excessive water, which contributes to the increment of thermal conductivity.^35,36^ The pure silica aerogel prepared by this method with a higher thermal conductivity when the ratio of diluted water glass is 5. So the thermal conductivity of the composite is higher when the *r* changes from 4 to 5.

**Table 2:** Effect of diluted ratio of water glass on the composite

Samples	Diluted ratio of water glass	Contact angle (*θ*)	Thermal conductivity (*λ*/W m^−1^ K^−1^)	Bulk density (kg m^−3^)
1	0 : 0	0	0.0670	245.2
2	1 : 1	137.4	0.0551	287.4
3	2 : 1	130.7	0.0542	298.7
4	3 : 1	136.2	0.0480	306.5
5	4 : 1	137.9	0.0564	290.6
6	5 : 1	132.7	0.0618	292.8

## 4. Conclusions

In this work, the silica aerogel/geopolymer composite was prepared by the impregnation with silica aerogel. SEM images show that the pores of geopolymer were filled with aerogel particles, directly indicating the successful preparation of this composite. Compared with that of porous geopolymer, the bulk density of composite increases from 235.2 to 306.5 kg m^−3^, the thermal conductivity decreases from 0.0670 to 0.0480 W m^−1^ K^−1^ and the maximum compressive strength increases from 0.45 to 0.79 MPa, when the diluted ratio of water glass is fixed at 3 : 1. These results show that the improved thermal conductivity and mechanical properties of sample can be obtained *via* the combination of geopolymer and silica aerogel, suggesting the possibility of prepared aerogel/geopolymer composite for insulating applications.

## Conflicts of interest

There are no conflicts to declare.

## Supplementary Material
